# Hidden voices: prevalence and risk factors for violence against women with disabilities in Nepal

**DOI:** 10.1186/s12889-015-1610-z

**Published:** 2015-03-18

**Authors:** Mahesh Puri, Geetanjali Misra, Sarah Hawkes

**Affiliations:** Centre for Research on Environment Health and Population Activities (CREHPA), Kathmandu, Nepal; CREA, New Delhi, India; University College London, London, UK

**Keywords:** Violence against women, Nepal, Disability, Inequity

## Abstract

**Background:**

There is an increasing body of evidence on the extent and predictors of violence against women in Nepal. However, much of the published research does not yet take into account additional features of marginalization and vulnerability suffered by some women – for example, women socially excluded on account of their disability. Critical gaps exist in empirical data on the extent, risk factors, access to care, socio-economic and health consequences of violence among women with disabilities in Nepal. This paper addresses some these gaps and aims to promote evidence-informed policy and programme responses in Nepal.

**Methods:**

We conducted a cross-sectional survey of 475 women with disability aged 16 years and above in three districts in Nepal. In-depth interviews with 12 women who reported violence in the survey were also carried out. Using multivariate statistical methods we estimated the prevalence and risk factors for violence experienced both over the past 12 months and lifetime.

**Results:**

Over the lifetime, 57.7% of women reported they had ever experienced violence, including emotional violence (55.2%); physical violence (34%); and sexual violence (21.5%). Over the preceding 12 months, 42% of women reported that they had experienced violence. Multivariate analysis showed that women with disabilities who were young, working in paid employment, and those who required permission from husbands/family to go to health centres or participate in community organizations were at increased risk of violence. Women experienced a range of negative outcomes from violence – including physical and emotional trauma. However, a majority of women did not seek care or redress from the health, justice or other sectors.

**Conclusions:**

Women in Nepal are at high risk of violence, often from members of their immediate family or local community. Rates of violence are higher in women with disability than among women in the general population. Tackling violence requires a comprehensive approach that addresses the root causes of women’s unequal position in society, and builds upon principles of equity and justice to ensure that all women are able to realize their rights to a life free from violence.

## Background

Nepal, home to 26.5 million people belonging to more than 120 different ethnic and caste groups, is a highly stratified and ethnically diversified society [1]. Social and power structures, institutionalized through a caste system, stratify individuals into unequal positions from and by birth. Exclusion and discrimination are perpetuated on the basis of caste, class, ethnicity, gender and even geographic location [2]. Among these multiple forms of inequity, people with disabilities are among the most deprived populations in Nepal, historically excluded from mainstream politics and socio-economic development [3]. People with disability are estimated to constitute 1.9% of the population [1] - a figure well below the global estimate of 15%, but perhaps reflecting differences in definitions used to classify disability.

Both physical and sexual violence are widespread against women and girls in Nepal. In the Demographic and Health Survey of 2011, 4.6% of girls aged 15–19 years reported experiencing sexual violence and one in five women aged 15–49 reported physical violence [4]; prevalence estimates are even higher in other surveys [5,6]. Women and girls suffer from a variety of forms of violence including accusations of witchcraft, violence associated with widowhood and dowry, and a large number of girls exploited within the South Asian sex trade.

A global meta-analysis estimated that people with disability have a 50% higher chance of suffering violence compared to non-disabled people, and among those with mental health problems the risks are even higher [7]; the same study noted that there is a dearth of evidence on the risk of violence suffered by people living with disabilities in low and middle income countries.

Evidence from Nepal is sparse but indicates that women living with disability are at increased risk of violence. A situation assessment conducted in 2001 documented 25% (N = 13,005 households) of people with disability who had been physically abused, and 25% had been mocked and taunted [8]. A study among 20 blind women in the capital, Kathmandu, found women faced with multiple forms of violence including physical violence, rape and incest [9]. A small study conducted among 35 women with disability found that 21 of them (60%) had experienced violence from their own family (including parents and husbands) – over half had experienced sexual violence, 1 in 5 had been raped, 60% reported being denied food and clothes or access to education or medical care, and 30% were economically exploited [10]. Despite the physical and emotional health risks associated with violence few women in the latter study reported seeking any health care subsequent to the violent episodes [9,11].

In 2009–2011 we conducted a multi-country study on violence against women who are excluded from ‘mainstream’ South Asian society, either on the grounds of disability, sexual orientation, or profession (sex work). This three-country study in Bangladesh, India and Nepal focused on marginalized women – those whose voices and experiences are rarely heard despite the gains made towards gender equality and the increasing media, social and academic attention being paid to violence against women in the region. In this paper we report on the results of a survey and in-depth qualitative research among women living with disability in Nepal – we focus on this group since for reasons of logistics and resources available in our study we have more data and evidence available from this group of women than from sex workers and lesbian women.

## Methods

**Figure 1:**
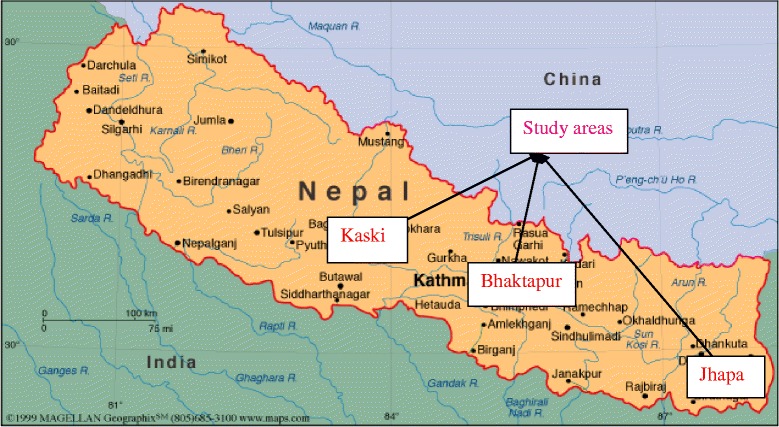
**Map of Nepal showing study areas.**

We conducted mixed method research, including cross-sectional quantitative surveys, in three districts of Nepal (Bhaktapur, Kaski and Jhapa) selected to represent regional and geographic diversity, a range of socio-economic backgrounds and the presence of non-governmental organizations (NGOs) working with disabled people. Figure 1 illustrates the locations of the three Districts where the study was conducted.

Nepal, a low income country in South Asia, has a mixed pattern of socio-economic development. While overall the country ranks 145th (out of 187) on the United Nations 2014 Human Development Index (HDI), the three Districts show some variation in development indicators – which include life expectancy, literacy, education, standard of living, and quality of life [12]. Jhapa, a District of over 800,000 is the easternmost district of Nepal and has a predominantly upper caste population. It has an overall HDI of 0.49 – compared to 0.54 for Nepal overall. Kaski and Bhaktapur have a more mixed ethnic make-up, and each has an HDI of 0.58. We included Districts both above and below the national HDI average in order to try and ensure a wide exposure to different contexts of socioeconomic development within the country.

In the quantitative survey, we have interviewed 475 women with disability aged 16 years and above, with either physical or sensory disabilities. Women with mental health problems and women with intellectual disabilities were excluded from the survey as we did not have the capacity (trained interviewers) to interview women with specialized needs.

A multi-staged sampling procedure was used to select disabled women for the individual interviews. In the first stage, a list of women with disabilities in each of the selected districts was prepared with the help of government/NGOs implementing community-based rehabilitation programmes. The sample size was distributed in the selected districts by using population (women with disability) proportion to size. In the second stage, all village development committees and municipalities were arranged in alphabetical order and the top seven village development committees or municipalities were selected. A total of 21 sites (15 village development committees and 6 municipalities) were chosen and the sample was allocated according to the population of the sampled areas. Finally, a list of women with disability for the selected village development committees/Municipalities was updated with the help of local key informants and then used to identify the households of these women who were invited to interview.

In the qualitative method, in-depth interviews with 12 women with disability were purposively selected from the survey respondents who reported any form of violence (physical, sexual, emotional), at least once in the past year during the individual interview. The sample size for in-depth interviews was guided by principles of diversification and data saturation – i.e. we stopped recruiting at the point of data saturation. In-depth interviews were planned at the design stage of the study and used in order to complement and supplement the findings from the quantitative survey.

All respondents gave informed consent to participate in both arms of the study. Interviews were conducted individually at a convenient location for the respondents, usually a private room within the woman’s house, by well-trained female Nepali interviewers, including one deaf interviewer who used sign language. Confidentiality of information was ensured by removing personal identification from the data and by securing and restricting access to all data and information. Interviewers orally provided the name and address of organizations that work with disabled people and deal with violence to all respondents. During the field study, one Nepali author of this article visited the study sites and supervised the interviewers to ensure interview quality and the respondent’s privacy. We used a structured questionnaire that included questions based on the WHO Multi-country Study on Women’s Health and Domestic Violence against Women [13], Questions were adapted to the local setting and study population. The core protocol and research instruments were approved by the Ethics committees at University College London and Center for Research on Environment Health and Population Activities.

Bi-variate and multivariate statistical analyses were conducted. Chi-squared tests were performed in order to explore bi-variate associations between respondent’s characteristics and experiences of violence. The bi-variate associations were tested on the variables that were important for theoretical reasons and on the basis of evidence from other countries. Multivariate analysis was then conducted in order to control for possible confounding. Due to the binary nature of the dependent variable (life time experience of violence or in the past 12 months), logistic regression was used. Two different models – one for identifying the factors associated with lifetime experiences of violence and another for recent experiences (in the last 12 months) were conducted. In Model 1, the dependent variable was whether or not the individual women reported any experiences of violence in her lifetime. In model 2, the dependent variable was whether or not the individual women reported experience of violence in the past 12 months preceding the interview. The independent variables were women’s age, caste/ethnicity, women’s level of education, number of living children, working status, exposure to the radio, decision-making power, able to visit health centre or any organisation without husband’s permission and whether she can refuse sex with her husband for any reasons. Selection of independent variables was guided by previous studies on gender-based violence. Only those variables that were significant or borderline significant in bi-variate analysis were included in multivariate analysis.

In-depth interviews were analyzed using content analysis. First, the IDIs were transcribed from audio-tape by field researchers and translated into English. Major themes from the transcripts were developed into codes for organizing and analyzing subsequent interviews. Using Atlas.ti (version 5) software, all the interviews were coded and the relevant quotations were extracted and interpreted and used to complement and supplement the quantitative findings.

We present the results of the quantitative survey supplemented by findings from the in-depth interviews as illustrative material giving further context and meaning to the quantitative results. Subheadings in the results sections were mainly derived from the quantitative data.

## Results

### Characteristics of respondents

We recruited 475 women, with a mean age of 44 years (age range 16–91 years),43% of whom were currently married and a third were unmarried at the time of interview. Among married women, 94% had at least one child. More than half of the women lived with their family members (52.6%) and 41.1% lived with their husbands. The largest proportion of women in the sample belonged to the upper caste group (43.4%) followed by the relatively advantaged indigenous groups (29.3%). Approximately 70%had less than primary level of education, and 74% were unemployed

A majority of women had a physical disability. A quarter of the women had visual impairment, one fifth were hearing impaired and one in ten had speech and hearing impairments. A quarter of respondents reported that they were disabled since birth, whereas one in five women said that they became disabled during infancy and childhood.

Women’s social capital was low: only a quarter of women were members of any type of community groups and a large majority (90.5%) were not associated with any organisations working with people with disability. In part, this may reflect the women’s restricted social mobility – 71% reported that they needed family member permission to join a community group, and 64% of women were not able to visit friends, relatives or a health centre without permission from their husbands and/or family members. Women’s access to mass media was low with less than one in five reading newspapers, less than half listening to the radio and only 5% with access to the Internet (Table 1).

**Table 1:** **Participants characteristics (N = 475)**

	**n**	**%**
**Age (years) mean, SD**	44.5	0.83
**Currently married**	202	42.5
**Number of living children**		
Unmarried and no child	155	32.6
Married but no child	29	6.1
1 child	39	8.2
2 children	71	14.9
3 and more children	181	38.1
**Less than primary education(i.e. <5 years education)**	330	69.5
Type of disability*		
Visual impairment	118	24.8
Physical disability	279	58.7
Deaf	99	20.8
Speech and hearing disability	50	10.5
**Age on onset of disability**		
By birth	120	25.3
<1 year	32	6.7
1-4 year	71	14.9
5 and over	252	53.1
**Living with**		
With family members	250	52.6
Husband	195	41.1
Alone	30	6.3
**Caste/ethnicity**		
Upper caste group	206	43.4
Relatively advantaged indigenous groups	139	29.3
Disadvantaged indigenous groups (*Janajatis*)	45	9.4
Non-touchable group (*Dalit*)	85	17.9
**Currently employed**	124	26.1
**Member of any community groups**	120	25.3
**Associated with any organization that work with women with disability**	45	9.5
**Not able to hold any group membership in the community**	335	70.5
**Not able to join any organization**	335	70.5
**Not able to go to health care centre/hospital**	307	64.6
**Not able to visit friends or relatives without permission**	306	64.4
**Never/rare exposure to newspapers**	385	81.0
**Never/rare exposure to radio**	271	57.1
**Never/rare exposure to television**	183	38.5
**Never/rare use of internet**	455	95.8

*Total number/percent may exceed 100 due to multiple disabilities.

### Prevalence and types of violence

Lifetime prevalence of violence was defined as the proportion of women who had experienced at least one act of physical, sexual or emotional violence by a current partner, former partner or non-partner(s) at any point in their lives. Fifty-eight percent of women had ever experienced violence including: emotional violence (55.2%); physical violence (34%); and sexual violence (21.5%). Violence in the past 12 months was reported by 42% of women: emotional violence (41.3%), physical (12.2%) and sexual violence (9.7%) – see Table 2.

**Table 2:** **Level and types of violence against women with disability (N = 475)**

**Types of violence**	**Lifetime**		**Within 12 months**	
	**n**	**%**	**n**	**%**
**Emotional violence.** ***Has anyone ever……***	**262**	**55.2**	**196**	**41.3**
Insulted you or made you feel bad about yourself	257	54.1	179	37.7
Belittled or humiliated you in front of other people	221	46.5	154	32.4
Done things to scare or intimidate you on purpose	113	23.8	66	13.9
Threatened to hurt you or someone you care about	68	14.3	55	11.6
Gave mental pressure to earn money	4	0.8	4	0.8
**Physical violence.** ***Has anyone ever……***	**162**	**34.1**	**58**	**12.2**
Slapped you or thrown something at you that could hurt you	125	26.3	31	6.5
Pushed you or shoved you or pulled your hair	107	22.5	36	7.6
Hit you with his fist or with something else that could hurt you	74	15.6	28	5.9
Kicked you, dragged you or beaten you up	61	12.8	14	2.9
Choked or burnt you on purpose	7	1.5	4	0.8
Threatened to use or actually used a gun, knife or other weapon against you	14	2.9	8	1.7
Thrown out from the house	11	2.3	7	1.5
**Sexual violence.** ***Has anyone ever…..***	**102**	**21.5**	**46**	**9.7**
Physically forced you to have sexual intercourse when you did not want to	81	17.1	26	5.5
Forced you to have sexual intercourse you did not want to because you were afraid of what your s/he might do	48	10.1	19	4.0
Force you to do something sexual that you found degrading or humiliating	36	7.5	22	4.6
Forced sexual activity like kissing, touching, masturbation, oral sex etc.	22	4.6	12	2.5
**Any type of violence**	**274**	**57.7**	**200**	**42.0**

The most common act of emotional violence was being insulted (54.1%) and being humiliated in front of other people (46.5%). About a quarter of respondents (23.8%) reported that the perpetrator scared or intimidated them deliberately. Moreover, one in seven (14.3%) had been threatened that they would be hurt or someone they cared about would be hurt.

Women in the in-depth interviews reported sustained and multiple forms of emotional abuse:

*“My father-in-law and mother-in-law are not happy with their son [her husband] because he has married a blind woman like him. They wanted their son to get married with sighted girl. They call me ‘beshya’ (prostitute) and say ‘you hang and die.”*

- **24 years, married, student, 15 years of education**

*“My father-in-law always used to take off his underwear and show his sex organ to me. He always used abusive words. He used to say ‘by looking at your ginger-like feet and hands early in the morning my entire day will be spoiled (bad luck)’. When I came back home from the hospital then my father-in-law kicked me and scolded me by saying ‘it would be better if you had died there.”*

**- 44 years, married, pickle seller, 10 years of education**

Of those who reported physical violence, 26.3% reported being slapped or having something thrown at them, women had been pushed or shoved or had their hair pulled (22.5%). One in six women also were hit with a fist or other object (15.6%) and more than one in ten reported being kicked, dragged or beaten (12.8%). A small number of women (2.3%) also reported being thrown out of the house.

In-depth interviews revealed similar stories - 9 out of 12 women had experienced different forms of physical violence.

*“He beats for very small matters. If he gets angry then he beats me a lot. Today, also he has beaten me”*

- **34 years, married, teacher, 12 years of education**

*“When I came back from the temple, my mother was locked in a room and was being tortured for two and half hours. When I asked what happened, my brother warned me not to enter there. When I went in, he clenched my hair and dragged me, slapped me and hit me with everything he could find”*

**- 48 years, unmarried, business, non-formal education**

Being physically forced to have sex was the most prevalent (17.1%) form of sexual violence, while being forced to do something humiliating or degrading (7.5%) and unwanted kissing, touching were less common (4.6%). Eleven out of 12 women interviewed in-depth reported experiencing sexual violence. One unmarried woman explained how she had been sexually abused by neighbours several times:

*“At the age of 12, an elderly brother from the neighborhood forced me for sex when I was alone at home. He called me in his room saying he will guide me in studies. He was staying in a rented room nearby and was in grade 9. When I reached there he forcefully take off my clothes and made me sleep in the bed and he forcefully had sex. When I cried he shut my mouth and did not let me cry and then he threatened to kill me if I told this to anyone. This kind of incident took place with me last year too by another man staying on rent behind our house. …… I have not shared this to anyone”*

**- 17 years old, unmarried, student, 10 years of education**

One woman with speech and hearing disability had been sterilized without her knowledge or consent. The physical and sexual violence from her disabled husband intensified after this incident. She explained:

*“…When I gave birth to my son, I was sterilized at the request of my mother-in-law. It was done without my knowledge…My husband having sex with me 4 to 5 times in a night saying I have to give birth to a baby girl. My husband beats me and scolds me when I say we cannot have baby because of sterilization…”*

**- 34 years old, married, housewife, illiterate**

### Risk factors for violence

Table 3 presents the percentage of disabled woman experiencing any type of violence in their lifetime, and in the past 12 months, by selected background characteristics. The results show that a woman’s age, number of living children, employment status and whether or not she perceives that a woman can refuse sex with her husband, were significantly associated with the experience of lifetime violence. Women aged 20–35 and 36–64 years were significantly more likely to report ever experiencing violence compared to women less than 20 or over 65 years. Women with disability who were working for cash incomes were more likely to report ever experience of violence compared to those who did not work for cash income. Women who believed that a woman can refuse sex with her husband were less likely report violence than those women who did not think women can refuse marital sex.

**Table 3:** **Percentage of women with disability reporting experience of violence by selected background characteristics and p-values from chi-square test**

**Characteristics**	**Lifetime**		**Past year**		**N**
	**N**	**%**	**N**	**%**	
**Women’s age**	**		ns		
<20	17	58.6	12	41.4	29
20-35	90	61.6	64	43.8	146
36-64	133	61.3	96	44.2	217
65 and over	34	41.0	28	33.7	83
**Caste/ethnicity**	ns		ns		
Upper caste group	120	58.3	88	42.7	206
Relatively advantaged indigenous groups	71	51.1	52	37.4	139
Disadvantaged indigenous groups	33	73.3	22	48.9	45
Non-touchable group (*Dalit*)	50	58.8	38	44.7	85
**Women’s education**	ns		ns		
Illiterate	128	57.1	94	42.0	224
Non formal education/1-5 years of schooling	76	62.8	57	47.1	121
6-10 years of schooling	44	55.0	31	38.8	80
Over 10 years	26	52.0	18	36.0	50
**Number of living children**	*		ns		
Unmarried, no child	80	51.6	55	35.5	155
Married, no child	15	51.7	12	41.4	29
1-2	75	68.2	52	47.3	110
3 and over	104	57.5	81	44.8	181
**Women’s occupation**	*		*		
Not working	180	54.1	128	38.4	333
Working for pay	94	66.2	72	50.7	142
**Wealth quintile**	ns		ns		
Lowest	62	65.3	42	44.2	95
Second	52	55.9	44	47.3	93
Middle	54	55.7	41	42.3	97
Fourth	55	57.9	39	41.1	95
Highest	51	53.7	34	35.8	95
**Exposure to radio**	ns		ns		
Never/not applicable	104	60.1	78	45.1	173
Rarely	48	49.0	33	33.7	98
Sometimes (2–3 days a week)	56	60.2	40	43.0	93
Almost everyday	66	59.5	49	44.1	111
**Disability status**	ns		ns		
Blind or deaf	66	55.9	55	46.6	118
Other physical disability but no blind	208	58.3	145	40.6	357
**Decision making**	ns		ns		
Self	56	59.6	38	40.4	94
Joint	4	33.3	4	33.3	12
Other family member	214	58.0	158	42.8	369
**Able to visit friends or relatives**	ns		ns		
Yes	93	55.0	64	37.9	169
No	181	59.2	136	44.4	306
**Able to visit health center or hospital**	ns		*		
Yes	93	55.4	59	35.1	168
No	181	59.0	141	45.9	307
**Able to visit any organization**	ns		**		
Yes	74	52.9	46	32.9	140
No	200	59.7	154	46.0	335
**Able to hold membership in the community group**	ns		ns		
Yes	80	57.1	53	37.9	140
No	194	57.9	147	43.9	335
**Agrees with justifying husband beating his wife for any reason**	ns		ns		
Agree	116	56.9	89	43.6	204
Disagree	158	58.3	111	41.0	271
**Whether a wife can refuse sex with her husband for any reason**	*		*		
Yes	49	71.0	37	53.6	69
No	225	55.4	163	40.1	406
**Total**	**274**	**57.7**	**200**	**42.1**	**475**

*P ≤ 0.05, **P ≤ 0.01, P ≤ 0.001, ns -Not significant, and + − p-value not available due to zero or near zero.

Compared to lifetime experiences of violence, only the working status of women, women’s mobility and perception about refusing sex with her husband was significantly associated with violence in the past 12 months.

A number of variables remained significantly associated with risk of violence on logistic regression - see Table 4. Young women aged below 20 years of age were 6.9 times (CI 2.2 – 21.6) more likely to experience lifetime violence compared with women aged 65 and above. Unmarried women were 55% (CI 0.2-0.8) less likely to face violence compared to those who were married and have had more than three children. Women who were not able to visit a health centre or any community organization without permission from their husband were more likely experience violence than those women who could visit without permission (aOR- 1.65 and CI- 1.2-2.7). Women who thought that a wife cannot refuse sex with her husband were 1.9 times more likely (CI- 1.1-.3.6) to experience violence compared with those who women thought that she can refuse sex with her husband for any reason. Conversely, women who made joint decisions with their husbands were significantly less likely to experience violence during their lifetime (aOR- 0.15 and CI −0.03-0.06). One other ‘protective’ factor was identified: women who were not working for cash income were significantly less likely to experience violence compared to women in paid employment (aOR – 0.56 and CI – 0.3-0.9).

**Table 4:** **Factors associated with violence, adjusted odds ratios and 95%confidence intervals from multivariate logistic regression**

**Characteristics**	**Model 1 (life time)**		**Model 2 (past year)**	
	**OR**	**C.I.**	**OR**	**C.I.**
**Women’s age**				
<20	6.94***	2.2 - 21.6	3.44*	1.1-10.6
20-35	4.34***	2.0 - 9.4	2.31*	1.1-4.9
36-64	2.92***	1.6 - 5.3	1.79*	0.9-3.2
65 and over (ref)	1.00	-	1.00	-
**Caste/ethnicity**				
Non-touchable group (*Dalit*)	0.63	0.4 - 1.1	0.75	0.4-1.3
Disadvantaged indigenous groups	1.69	0.8 - 3.6	1.11	0.6-2.2
Relatively advantaged indigenous group	0.75	0.5 - 1.2	0.78	0.5-2.2
Upper caste group (Ref)	1.00	-	1.00	-
**Women’s education**				
Illiterate	1.69	0.7 - 3.9	1.23	0.5-2.8
Non formal education/1-5 years of schooling	1.50	0.7 - 3.3	1.35	0.6-2.9
6-10 years of schooling	1.10	0.5 - 2.4	1.01	0.5-2.2
SLC and above (Ref)	1.00	-	1.00	-
**Number of living children**				
Unmarried, no child	0.45**	0.2 - 0.8	0.44**	0.2-0.8
Married, no child	0.57	0.2 - 1.4	0.72	0.3-1.7
1-2	1.434	0.8 - 2.5	1.00	0.6-1.7
3 and more (Ref)	1.00	-	1.00	-
**Women’s working status**				
Not working for cash income	0.56*	0.3 - 0.9	0.51**	0.3-0.8
Working (Ref)	1.00	-	1.00	
**Exposure to radio**				
Never//Rarely/not applicable	1.46	0.9 - 2.2	1.41	0.9-2.1
Sometimes/Almost everyday (Ref)	1.00	-	1.00	-
**Decision making power**				
Other family member	0.90	0.5 - 1.6	0.93	0.5-1.7
Joint	0.15**	0.03 - 0.6	0.41	0.1-1.6
Self (ref)	1.00	-	1.00	-
**Able to visit health center or any organization**				
No	1.65*	1.0 - 2.7	2.20**	1.3-3.6
Yes (Ref)	1.00	-	1.00	-
**Women’s perception on whether she can refuse sex with her husband**				
No	1.98*	1.1 - 3.6	1.66	0.9-2.9
Yes (Ref)	1.00	-	1.00	-

*P ≤ 0.05, **P ≤ 0.01, ***P ≤ 0.001.

Overall, no major differences were observed in the findings between Model 1 (lifetime experience of violence) and Model 2 (violence in the past 12 months).

### Perceived reasons for violence

The large majority of interviewees (80%) believed that their disability was a major cause of violence against them. Women also believed that an inability to work (74.1%), stigma (34.7%) and poverty (34.7%) were reasons for violence against women with disability.

*“I don’t have strong feet. I cannot run if something happens. When one is disabled, one cannot stop the problems. Even if we try, we are more engulfed by problem. When we suffer, we do not get support from anywhere. People look at us with hatred”*

- **20 years, married, housewife, illiterate**

### Perpetrators

Over half (58%) of women experienced violence from family members, neighbors (52.6%), intimate partners (39.1%), and friends (8%). Strangers were a less frequently perpetrators of violence (12.8%). Sexual violence was most likely to be perpetrated by husbands/intimate partners, while family members and neighbors were the main perpetrators for emotional and physical violence. In-depth interviews gave some insights into the types of violence, attitudes towards women with disability, relationship with families and highlighted the specific experiences of gender discrimination in Nepali society. For example, a woman who developed a progressive deterioration in her manual functioning described the impact on her quality of living and her sense of self-worth in relation to her fully-functioning peers and relatives said:

*“Since I am not able to do lot of work in the house so, everyone dominates and scolds me. I can’t wash clothes, my hands doesn’t function well. No one looks after me when I fall sick. Few days ago, when I was sick no one gave me a glass of hot water. My husband didn’t care me. If something happens to me then my brothers-in-law curse me and say “Why do disabled people live? It is better for you to die.”*

**- 44 years, married, pickle seller, 10 years of education**

### Consequences of violence

Women reported a variety of psychological and physical problems associated with violence – see Table 5.

**Table 5:** **Self-reported negative consequences of violence**

**Consequences**	**N**	**%**
**Experience of psychological problems**		
Yes	249	91.2
No	24	8.8
**Total**	**273**	**100.0**
Types of psychological problems faced*		
Fear	146	58.6
Tension	195	78.3
Depression	134	53.8
Suicidal feeling	64	25.7
Tried to take your own life	5	2.0
Worried	8	3.2
Left home	11	4.4
**Total**	**249**	*
**Experience of physical problems**		
Yes	51	18.7
No	222	81.3
**Total**	**273**	**100.0**
Types of physical problems faced*		
Cut	17	33.3
Sprain	10	19.6
Burn	1	2.0
Broken bone	1	2.0
Broken head	6	11.8
Backache/ headache/ bodyache/ swollen gums/ swollen cheeks	16	31.4
Broken Ear Drum/earache	4	7.8
Nose bleeding	1	2.0
Had been unconscious	1	2.0
Uterine injury	1	2.0
**Total**	**51**	*
**Experience of reproductive health related problems**		
Yes	21	7.7
No	252	92.3
**Total**	**273**	**100.0**
Types of reproductive health problems faced*		
Pregnancy loss	2	9.5
Heavy bleeding	11	52.4
Severe abdominal pain	16	76.2
Uterus prolapsed/ uterine injury	4	19.0
Itching/ pain/burn in sex organ	1	4.8
**Total**	**21**	*

*Percentage total may exceed 100 due to multiple responses.

*“I don’t know what to do… I have too many injuries. Look here …yesterday he beat me and my hand is swollen [showing her hand]. My heart is filled with pain and agony. Sometimes, I want to die by jumping out of this window. Brothers-in-law and neighbors don’t speak with me. My husband only needs alcohol. I have tension both day and night. Due to tension I have developed high blood pressure. No one is there to support me”*

- **44 years, married, pickle seller, 10 years of education**

*“On that day he tied my both legs with ropes and made me kneel down and face towards the ground and had anal sex. It was intolerable so I screamed, but he closed my mouth and beat me very badly. I was not able to tolerate it so I went to die at midnight in Begnas lake but the neighbors came to know about it and stopped me”*

- **29 years old, married, business, non-formal education**

Two women reported being disabled as a result of physical violence inflicted by their husband.

*“My sister’s son had fallen ill and I went to see him. The child was sick so I reached home late. That day my husband and his family didn’t let me enter the house and also didn’t give me anything to eat and beat me up. He blamed me for going to another man for sex since he is not able satisfy me. At that night he forced me and put his penis into my mouth. When I made a lot of noise he beat me in head with a door stand (big stick used to lock the door from inside), I had to operate my eyes because of that.”*

**- 34 years, divorced, unemployed, 10 years of education**

Five interviewees reported reproductive health problems consequent to violence – including severe abdominal pain, burning sensation, and heavy bleeding during periods. A 24 year old, illiterate blind woman was raped by a blind man who promised to marry her. She became pregnant and her family decided to abort the child due to fear of social stigma and humiliation. She reported:

*“ The first time he took off my suruwal (pant) but I protested and he forcefully got on top of me and inserted it (penis). I hit him with a stick and cried but there was no one besides the two of us. Nobody heard me… He said that he loves me and marry me. I was confused for a while and I believed him. I didn’t tell this to anyone but that mora (stupid guy) left me behind. I got pregnant and had an abortion. I had no other option”.*

**- 23 years, unmarried, unemployed, illiterate**

*“As long as I am living, some kind of fear always disturbed me that the others might find out about the rape. I also started getting more pain at my lower abdomen during my period. I never had pain before. I also feel dizzy from time and bleed heavily during my menstruation. When I recall all these things, sometimes I have a lot of mental torture and feel like dying.”*

**- 17 years, unmarried, student, 9 years of education**

### Seeking care and support

The majority of women (60%) who had experienced violence did not seek care or support – either from named institutions or more informal support networks of friends and family members.

Among those women who did seek care, there was an association between perpetrator and care-seeking: 77.6% of women who had experienced violence from their intimate partners did not seek care or support compared to 52.5% among women who suffered a similar type of violence from someone other than an intimate partner. Reasons for not seeking care included not knowing where to report (21.9%) and physical access problems (15.6%) in reaching institutional support.

Three interviewees who had faced violence from their intimate partner or from their family members reported the situation to the police or other authority figures – not always with positive results:

*“The policemen scolded me for crying. Then, my brother came and a lot of others who supported him also came. I did not have anyone. My brother confessed about beating me but he did not disclose the reason for doing so. Then the policemen said that the people from maiti (maternal home) have the right to beat the Cheli (daughters and sisters).”*

**- 48 years, unmarried, petty business, informal education**

*“We do not receive good service; the government officers are hungry for money. If you have money then it will be good to go to the government officers but without money nothing will happen. …When I went for my divorce I couldn’t find a lawyer to fight my case because the lawyer straight away asked for money…”*

**- 34 years, divorced, unemployed, 10 years of education**

However, in-depth interviews revealed that some women do find support. Three women reported to a mother’s group and received a positive experience from them:

*“Nowadays he has not beaten me. The mother’s group had written an agreement and made him sign on it. So, he is scared to hit me.”*

**- 29 years, married, petty business, informal education**

## Discussion

Violence against women with disabilities lies at the intersection of gender and disability, and is fostered by a culture that devalues, and systemically disempowers, both women and disabled people. Women face diverse forms of violence throughout their lives mainly due to the subordinate space they occupy in society at large and, as noted in our findings, within the family in particular. Women who are marginalised or excluded from mainstream society may face particular risks of violence – and a lower access to care and support. Our study among women living with disability in Nepal has found high levels of different types of violence and a variety of barriers to effective support and redress.

This is the first large scale study of the intersection between gender, disability and violence against women in Nepal. The survey results have been given context and meaning through in-depth interviews which serve to illustrate the realities of women living with disability and experiencing violence. Despite the relatively large sample size, our study had limitations that may have affected findings. Firstly, we were unable to include women with intellectual and mental disabilities – whose experiences of violence may be different to the women that we interviewed. Secondly, we relied upon an initial listing from NGOs and Government services. Women who are particularly highly marginalized and ‘hidden’ due, for example, to extreme gatekeeper pressures, may not have been included in our listings. Thirdly, respondents’ concerns about safety and the presence of a perpetrator of violence at home may have led to underreporting. However, we aimed to minimize this as much as possible by guaranteeing respondents’ privacy and confidentiality and ensuring that no one in the household was aware of the contents of the questionnaire. Finally, the study was cross-sectional and thus temporal sequence and causation cannot be established.

The levels of violence reported by women in this survey are higher than those reported by women across the general population in the most recent Demographic and Health Survey of Nepal, and other population-based surveys of violence against women [4,5]. We found an increased risk (on logistic regression) among some groups of women – those who were young, those who required permission from husbands/family to go to health centres or participate in community organisations, and women who perceived they did not have the right to refuse sex with their husbands. These findings may represent women with lower levels of autonomy, for example on account of age or perhaps because of disability status. Some women were noted to have lower risks of violence – women who were unmarried, women who were involved in joint decision-making with their husbands, and women not working for a cash income. The association with marital status is perhaps a reflection that the most commonly listed group of perpetrators of violence, particularly sexual violence, was husbands. However, the finding of an increased risk of violence among women in paid employment needs further exploration. This may reflect the increased willingness of these women to report their experience of violence, i.e. these may be women with an increased autonomy and possibly a stronger sense of gender equality. It may also represent the exposure of women to violence when they are outside of their home – for example in public and working spaces. Similar suggestions regarding women’s willingness to report violence were made from a recent Europe-wide survey of violence against women across 28 countries [14].

The perpetrators of violence were usually known to the women – members of their own family, or close community. This may have added to the reluctance of women to seek care either in the health, justice or other sectors. In-depth interviews illustrated some of the reasons for lack of care seeking, including stigma, shame, fear of further violence, low knowledge about appropriate care services, lack of physical access to services, and a lack of belief/trust that public sector services would provide the care and support they needed.

The legal and policy environment in Nepal is generally supportive for people with disability, but still contains some laws which may both perpetrate stigma and preclude redress for

violence and discrimination suffered by women with disability. For example, the National Code 1964 permits a second (concurrent) marriage if the spouse becomes blind or ‘crippled’ [15]. Though this provision applies to both men and women, it is generally exercised more by men than women. The law also sets out to protect women with disability: under the 11th amendment of *Naya Muluki Ain* (Country Code), any perpetrator (or perpetrators in the case of gang rape) of sexual violence against “pregnant, disabled or handicapped women” will receive an additional 5 years added to their sentence [15]. At the international level the Government has ratified a number of international conventions and treaties that, in theory, promote gender equality for all women, and, in particular, ensure that the rights of women (and men) with disability are promoted and protected– for example, the Convention on the Elimination of Discrimination against Women was ratified in 1991, and the Convention on the Rights of Persons with Disabilities in 2010. However, our study has highlighted a number of areas where women’s rights, including the rights of women with disabilities, are not being fully ensured. Women are being subjected to violence in wider society, and, more frequently in one of the places where they should feel most safe: their own homes.

Women with disability are unable to participate fully in society and access the services they require and to which they are entitled. Women spoke to us of not trusting police and justice sectors to protect them, and of being treated inadequately by others in care-giving positions. Moreover, one in six women indicated that they could not seek care as they could not physically access buildings such as police stations or health centres. The Government has promoted a network of “rehabilitation centres” for women who have suffered from violence, but few of these exist to serve women with a disability. Promoting equal-access services for women with disability who have suffered violence would be an important first step in promoting access to justice and health care. Ensuring that service providers are held accountable for mandated service delivery within a respectful and rights-based environment is also required.

## Conclusions

We believe that there are a number of changes that need to be in place for the situation of women with disability to improve. First and foremost, Nepal is a patriarchal gender-unequal society that currently ranks globally at position 102 (out of 148 ranked countries) on the United Nations Development Programme assessment of Gender Inequality [16]. Addressing the root causes of violence against all women in Nepal requires tackling the fundamental causes of women’s unequal position in Nepali society. While social and health sectors can provide interventions and services to address the needs (regardless of disability, age, caste, sexual orientation etc.) of women who have suffered violence, of fundamental importance is the need to address the social (and political and cultural) determinants of violence. Addressing such determinants will require a multi-sectoral, multi-intervention approach that has political and social support. The Nepali constitution is being rewritten for the first time in 23 years, thus affording the opportunity to promote concepts of equality and equity as fundamental principles for everyone. Recent changes to national laws – both promoting gender equality and protection against violence – may help to provide an environment in which women are more able to claim their rights as equals. However, despite the existence of a supportive legal environment, there is a notable lack of accountability for implementation.

Holding Governments to account for the services to which people are entitled is one important aspect of ensuring that women receive the care and support to which they are entitled. However, of equal importance will be to continue to focus on steps to ensure that all women (whether living with disability or not) can live as equal members of a socially just society – a goal that requires change not just within health, justice and social care sectors, but a fundamental shift in political and cultural norms within Nepali society.

## References

[1] Central Bureau of Statistics (2012). National Population and Housing Census 2011, Volume 02, 2011.

[2] DFID/World Bank: Unequal citizens (2006). Gender, Caste and Ethnic Exclusion in Nepal. Summary Report.

[3] Norwegian Agency for Development Cooperation (2014). Mainstreaming disability in the new development paradigm: Evaluation of Norwegian support to promote the rights of persons with disabilities- Nepal country study.

[4] Ministry of Health and Population, New ERA, ICF International Inc (2012). Nepal Demographic and Health Survey 2011.

[5] Puri M, Forst M, Tamang J, Lamichhnae P, Shah I (2012). Theprevalenceanddeterminantsof sexual violenceagainstyoungmarriedwomenbyhusbands in rural Nepal. BMC Res Notes.

[6] Puri M, Tyynela J, Chain E, Armytage L, Adhikari U, Giri R (2013). A study on advancing justice sector reform to address discrimination and violence against women in four selected districts of Nepal.

[7] Hughes K, Bellis MA, Jones L, Wood S, Bates G, Eckley L (2012). Prevalence and risk of violence against adults with disabilities: a systematic review and meta-analysis of observational studies. Lancet.

[8] New Era (2001). A Situation Analysis of Disability in Nepal.

[9] Aryal N (2004). Silent Screams: A Study on the Sexual Violence Against Blind Women of the Kathmandu Valley.

[10] National Disabled Women Association (2008). Social Inclusion, livelihood and violence against women. Nepal Women with disability's Association.

[11] Watts C, Zimmerman C (2002). Violence against women: global scope and magnitude. Lancet.

[12] United Nations Development Programme/Government of Nepal (2014). Nepal Human Development Report 2014: Beyond Geography, Unlocking Human Capital.

[13] World Health Organization (2005). WHO Multi-Country Study on Women’s Health and Domestic Violence Against Women.

[14] European Agency for Fundamental Rights. Violence Against Women: an EU-wide survey. Accessible at: http://fra.europa.eu/sites/default/files/fra-2014-vaw-survey-at-a-glance-apr14_en.pdf Accessed on 28.05.2014

[15] Ministry of Women Child and Social Welfare and Forum for Women and Developmen (1995). *‘Mahilako Bibaha Tatha Sambandha Bichhedh Ko Adhikar’* (Marriage and Right of Divorce of women).

[16] United Nations Development Programme. Gender inequality Index- UNDP open data. Available from https://data.undp.org/dataset/Table-4-Gender-Inequality-Index/pq34-nwq7, accessed on 29.05.2014

